# Theoretical and Experimental Study on Wide Range Optical Fiber Turbine Flow Sensor

**DOI:** 10.3390/s16071095

**Published:** 2016-07-15

**Authors:** Yuhuan Du, Yingqing Guo

**Affiliations:** School of Power and Energy, Northwestern Polytechnical University, Xi’an 710129, China; duyuhuan@mail.nwpu.edu.cn

**Keywords:** FFT, double-circle-coaxial fiber, turbine flow sensor model, wide range, piecewise linear, TH814, TP27

## Abstract

In this paper, a novel fiber turbine flow sensor was proposed and demonstrated for liquid measurement with optical fiber, using light intensity modulation to measure the turbine rotational speed for converting to flow rate. The double-circle-coaxial (DCC) fiber probe was introduced in frequency measurement for the first time. Through the divided ratio of two rings light intensity, the interference in light signals acquisition can be eliminated. To predict the characteristics between the output frequency and flow in the nonlinear range, the turbine flow sensor model was built. Via analyzing the characteristics of turbine flow sensor, piecewise linear equations were achieved in expanding the flow measurement range. Furthermore, the experimental verification was tested. The results showed that the flow range ratio of DN20 turbine flow sensor was improved 2.9 times after using piecewise linear in the nonlinear range. Therefore, combining the DCC fiber sensor and piecewise linear method, it can be developed into a strong anti-electromagnetic interference(anti-EMI) and wide range fiber turbine flowmeter.

## 1. Introduction

Flow rate measurement is of great importance to a wide range of industries including oil and gas, chemical and textiles, food production and the pharmaceutical industry [1]. As a widely used flowmeter in recent years, electromagnetic turbine flowmeter (EMTFM) plays an important role in the liquid measurement. Based on the principle of electromagnetism, the rotational speed of turbine in the pipe is detected and then converted to flow rate [2]. However, one of the disadvantages of this type of flowmeter is its limited range ratio, which causes some inconvenience in large flow measurement conditionthat’s because several different caliber turbine flowmeters are simultaneously needed to coordinate; in addition, electromagnetic flowmeter is vulnerable to electromagnetic interference(EMI), which limits its application in a number of applied occasions.

Fiber optic systems are immune to EMI and have large bandwidth, good accuracy and sensitivity [3]. The valuable characteristics of flowmeters based on fiber optics make the exploitation attractive and motivate the growing scientific interest in the technology [4]. As fiber-optic sensor technology has developed, more and more new flowmeters have been proposed combining the traditional flowmeter concepts and the various fiber-optic technologies [5]. Many types of optical fiber have been explored in flow measurement field. R. P. Hu [6] proposed a new type of optic flowmeter system based on the bending loss of an optic fiber. A. Vijayan [7] explored the effective use of optical fiber for air flow measurement. He proved the feasibility of new concept of hinge sensor for flow. The novel concept of a hinge joint was introduced in optical fiber, by which the macro bending that was produced was localized. J. Lim [8] presented some preliminary results of the DP flow sensor, which employed the two optical FBGs on a thin diaphragm. The two sides of the diaphragm were connected to sense DP across an orifice plate located in the fluid pipe. Y. Zhao [9] proposed a target type flowmeter based on a differential fiber Bragg grating sensor. The preliminary experimental results indicated that this sensor can be used to measure the fluid flow with the flow-rate from 0 to 1000 cm^3^/s, and the cross-sensitive problem of FBG sensors is effectively solved by the differential method. P. Lu [10] proposed a new fiber-optic sensor system consisting of a fiber Bragg grating cantilever as a transducer and demonstrated to realize simultaneous measurement of fluid flow rate and direction. E. Schena [3] proposed a novel fiber optic macro-bend based gas flowmeter for low flow rates. The fiber tip undergoes a deflection in the flow, acting like a cantilever. The consequent displacement of light spot center was monitored by the QD generating four unbalanced photocurrents which were a function of the fiber tip position. P. Saccomandi [11] designed and realized a new optoelectronic flow sensor to detect bidirectional volumetric air flow-rate.

In reference [12], the authors proposed using reflective optical fiber to measure flow rate, but the practical products have not been yet appeared up to now. This article develops a double-circle-coaxial(DCC) light intensity modulation fiber optic turbine flowmeter, which has more broad application prospects in comparison with EMTFM, because it has high sensitivity [13], strong anti-EMI, large range ratio [14,15] and remote transmission, etc.

## 2. Detecting Mechanism

**Figure 1:**
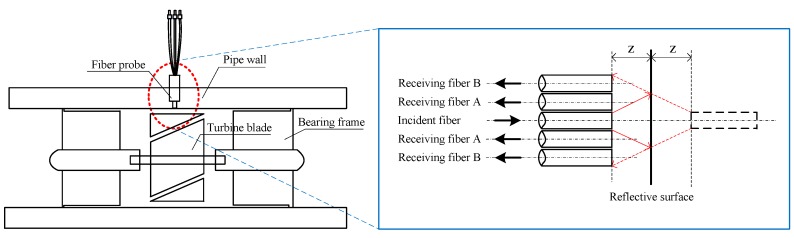
Fiber turbine flow sensor operating principle.

As is shown in Figure 1, a fiber probe is installed in the pipe wall vertically by puncturing and sealing, which ensures the incident light is fully irradiated onto the turbine blades. The light intensity received by receiving fiber changes when the turbine rotates, thus the rotational speed of the turbine can be detected and converted into flow rate. The light coupling principle of optical fiber sensor is as follows: according to geometrical optics, assume the displacement between the head face of optical fiber and the object to be measured is *z*, so there will be a virtual image of launching fiber away from the measured object surface in the distance of *z*. After the light from the launching fiber is reflected by the reflector, part or all of the light enters the receiving fiber. When the displacement *z* changes, the area covered by the spot changes accordingly [16,17,18], thus, the total amount of reflected light received by the receiving fiber will also change, i.e., the transmission of light is modulated.

### 2.1. Design of the Fiber Probe

**Figure 2:**
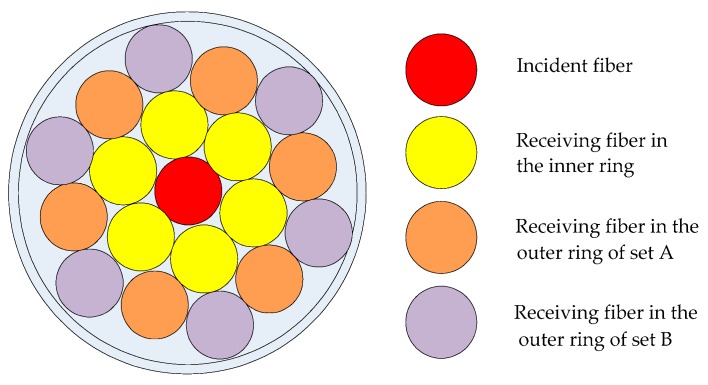
The double-circle-coaxial (DCC) fiber probe schematic diagram.

The DCC fiber structure is introduced in the turbine flow sensor, which includes the structure of the fiber probe and the arrangement of the fibers, as can be seen in Figure 2. Among them, the incident fiber is the one in the middle, and the others are the receiving fibers, which have spaces in two rings. The inner ring receiving fiber consists of 6 fibers, while the outer ring is made up of 12 roots of fibers. According to the distance between the input fiber and receiving fiber, the roots of 12 fibers in the outer ring are distinguished into two groups: set A and set B. Since the light received in two rings of receiving fiber is from the same emitting light source and reflecting surface, light intensity fluctuations of light source and the surface reflectivity are the same. Therefore, the two rings of the receiving fiber affected by the environmental factors are the same, such as fiber bending, light intensity fluctuation influenced by temperature, pressure and so on. That is to say, the two rings of DCC receiving fibers have the same sensitivity to the slight variations of light source power and the reflecting surface. Therefore, by dividing the light intensity reflected in the outer ring and the inner ring, the above interference can be eliminated. For this reason, the DCC fiber flow sensor has strong anti-interference and accuracy.

In terms of the structure of turbine flow sensor studied in this article, modeling simulation and optimization were undertaken [19,20]. Ultimately, the optimal design parameters of DCC fiber probe were determined as shown in Table 1.

### 2.2. Analysis of Signal Processing Method

Frequency measurement usually has two methods in application: the counting in time-domain and the transformation in frequency-domain. Counting is the most widely researched method [21], its essence is the measurement of time interval, and it mainly uses the direct frequency measurement method, the cycle measurement and multi cycle synchronous frequency measurement method, etc. For the time domain frequency measurement method, on the one hand, the quality of the signal requirements is higher. Generally, signals are required to become a standard pulse signal, which increases the difficulty of hardware development. On the other hand, in order to improve the measurement accuracy, the measurement time is increased, which actually decreases the real-time performance of the system. Therefore, it is difficult to accurately measure the weak signal with noise and interference by the time domain counting method. The frequency domain measurement method generally converts the signal into the frequency domain by the Fast Fourier Transformation (FFT), which is suitable for the weak signal with noise. It transforms the time domain signals into the frequency domain, and the peak value of the signal spectrum is the frequency value of the current signal. For the fiber optic sensors studied in this paper, the output signals include the inner ring and outer ring optical signals. Digital signal processing is needed in order to calculate the ratio of these two signals. That is to say, the information processing is done by software instead of hardware. In addition, due to the blades being thin, the light intensity signal is faint, so that the signal processing of the fiber turbine flowmeter is more suitable for frequency-domain transformation.

Fourier transformation is the core of the turbine rotation frequency measurement by using spectrum analysis [22]. According to the definition of Fourier Transformation, under a certain frequency *w*, frequency of the signal amplitude spectrum function reflects the size of the relative magnitude of the sinusoidal component of *w*. So in the turbine rotational frequency measurement, collecting sufficient sample signals is the first step, through frequency domain transformation to find out the frequency component of the maximum amplitude spectrum. The frequency component is the fundamental frequency of the periodic signal, namely the turbine rotational frequency, and the other frequency components are harmonic components. A commonly used algorithm for transforming the discrete sampling signal from the time domain to the frequency domain is the Discrete Fourier Transformation (DFT). Due to the lower efficiency of direct use of the DFT algorithm, a fast algorithm is generally used in the application, which is the FFT.

## 3. Research on Range Ratio of Turbine Flowmeter

Every turbine flowmeter has its measuring range. The upper limit of the range is decided by the turbine rotational speed. If the flow rate is too fast, it will cause the life of the turbine bearings to reduce or even the impellers and the bearings to become damaged; the lower limit of range ties to the accuracy of measurement. Therefore, in order to improve the flow measurement range ratio without changing the rotational turbine, the lower limit of range should be expanded.

**Figure 3:**
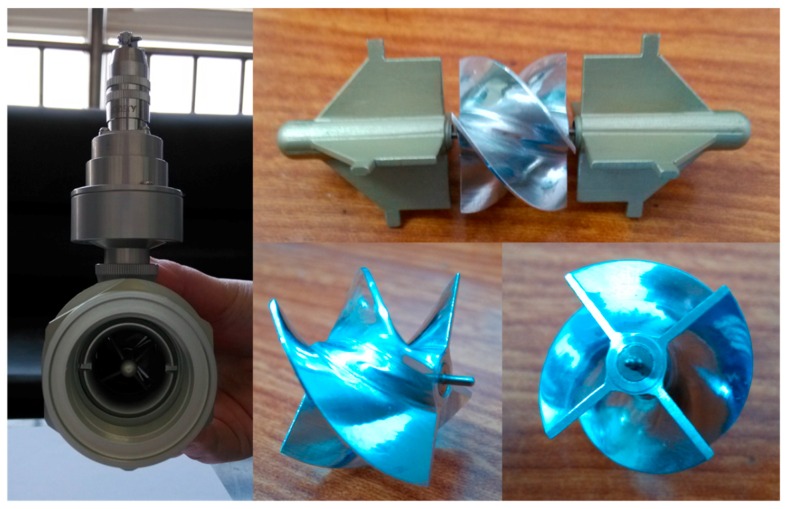
The DN20 turbine flow sensor picture.

In this paper, the DN20 turbine flow sensor is similar to the research object illustrated in Figure 3. Measuring range has been given by the manufacturer, while the data below the range was needed. Without precise calibration equipment in laboratory, in order to obtain the variation law of turbine flow sensor below the range, some contributions were made on the model of the turbine flow sensor. MATLAB/Simulink, with its graphical interface, is convenient to operate, debug and modify, and it has a dynamic display. Therefore, the mathematical model of the turbine flow sensor was built in MATLAB/Simulink.

### 3.1. Mathematical Model of Turbine Flow Sensor [23,24]

Assume the installation angle of turbine blades is θ, according to the outlet velocity triangle, the turbine rotational speed can be written as

(1) $$\omega =\frac{q}{A\bar{r}}\tan\theta (1-\frac{\tan\alpha }{\tan\theta })$$

where α is the exit angle, which is between the direction of the leaving absolute velocity from the turbine blade and the axes; $\bar{r}$ is the root mean square radius of the blade hub radius $R_{t}$ to the blade tip radius $R_{h}$, $\bar{r}$ can be expressed as follows

(2) $$\bar{r}=\sqrt{\frac{R_{h}^{2}+R_{t}^{2}}{2}}$$

Instrument constant $k_{0}$ can be computed by ω=2πf/N

(3) $$k_{0}=\frac{f}{q}=\frac{N\tan\theta }{2\pi \bar{r}A}(1-\frac{\tan\alpha }{\tan\theta })$$

In Equation (3), N is the number of blades.

In the ideal case, that is, resistance torque does not exist, α=0, so there is

(4) $$k_{0}=\frac{N\tan\theta }{2\pi \bar{r}A}=const$$

In Equation (4), $k_{0}$ is the ideal instrument constant, and it is only related to the structural parameters of the turbine flowmeter. When a variety of resistance torques exists, Equation (8) can be shown as

(5) $$T_{d}=T_{b}+T_{w}+T_{t}+T_{h}+T_{i}+T_{m}=\rho q^{2}(\frac{\bar{r}}{A})\tan\alpha$$

In Equation (5), $T_{d}$ is the blade driving torque, $T_{b}$ is the viscous friction resistance torque between shaft and bearing, $T_{h}$ is the viscous frictional resistance torque on the circumferential surface of the wheel hub, $T_{t}$ is the viscous frictional resistance torque between the blade tip and the inner wall of the sensor shell, $T_{w}$ is the viscous frictional resistance torque on the surface of the wheel hub, $T_{i}$ is blade surface viscous friction torque, $T_{m}$ is the sum of the electromagnetic resistance torque and the additional resistance torque.

Substituting Equation (5) into Equation (3) gives

(6) $$K=\frac{N\tan\theta }{2\pi \bar{r}A}(1-\frac{T_{b}+T_{w}+T_{t}+T_{h}+T_{i}+T_{m}}{\rho q^{2}\frac{\bar{r}}{A}\tan\theta })$$

According to Equation (6), when the resistance torque calculation formulas are known, the actual instrument constant values can be estimated according to the sensor structure parameters. The detailed calculation methods of each torque are shown as follows:

(1) The viscous frictional resistance torque between shaft and bearing $T_{b}$
  (7) $$T_{b}=\frac{4\pi R_{1}^{2}R_{2}^{2}}{R_{2}^{2}-R_{1}^{2}}L_{b}\rho \nu \omega$$
  $R_{1}$ is the shaft radius; $R_{2}$ is the bearing radius; $L_{b}$ is the total length of the friction region between the shaft and the bearing; the UOM of these parameters above is *m*.
(2) The viscous frictional resistance torque on the circumferential surface of the wheel hub $T_{h}$
  (8) $$\begin{array}{l} T_{h}=\frac{1}{2}\rho V_{zh}^{2}A_{h}R_{h}C_{h}\frac{\tan\beta_{\infty h}}{\cos\beta_{\infty h}}\\ A_{h}=2\pi R_{h}L_{h}-Nt_{bh}c_{h}\\ \begin{array}{ll} C_{h}=1.328R_{eh}^{-\frac{1}{2}} & \left(R_{eh}<2.5\times 10^{5}\right)\\ C_{h}=0.074R_{eh}^{-0.2} & \left(R_{eh}>2.5\times 10^{5}\right) \end{array}|\;R_{eh}=\frac{U_{\infty ch}c_{h}}{\nu } \end{array}$$
  where $V_{zh}$ is the fluid axial velocity in the hub position; $t_{bh}$ is the blade thickness in hub position; $U_{\infty ch}$ is the average relative velocity in hub, which approximately equals to $V_{zh}$; $\beta_{\infty h}$ is the angle of the relative velocity between the inlet and outlet, which approximately equals the setting angle of blade θ.
(3) The viscous frictional resistance torque between the blade tip and the inner pipe wall $T_{t}$, in which, $t_{bt}$ is the thickness on the blade tip.
  (9) Tt=12ρ(ωRt)2ctRttbtNCDtCDt=2/Ret(Ret<1000)CDt=0.016/Ret0.25(Ret>1000)
(4) The viscous frictional resistance torque on the surface of the wheel hub $T_{w}$
  (10) $$\begin{array}{l} T_{w}=\frac{1}{2}\rho \omega^{2}R_{h}^{5}C_{M}\\ \begin{array}{ll} C_{M}=3.87R_{\omega }^{-0.5} & \left(R_{w}<3\times 10^{5}\right)\\ C_{M}=0.146R_{\omega }^{-0.2} & \left(R_{w}>3\times 10^{5}\right) \end{array}|\;R_{w}=\frac{R_{h}^{2}\omega }{\nu } \end{array}$$
(5) The blade surface viscous friction torque $T_{i}$
  (11) Ti=12ρVzh2AtRhfhtanβ∞hcosβ∞hAt=2Nctrfh=1.328Rel−12(Rel<200)Ch=0.455/(logRel)2.58(Rel>200)| Rel=U∞chctν
  In Equation (11), $c_{t}$ is the chord length at the top of blade.
(6) The electromagnetic resistance torque and the additional resistance torque $T_{m}$

The electromagnetic signal detector has an impact on the impeller rotation, this effect is mainly due to an additional drag torque generated by electromagnetic signal detector which is independent of the impeller rotational speed [25]. The torque is codetermined by the structure of electromagnetic signal detector and wheel bearing lubrication effect. When the turbine shaft starts rotating, it has not only dry friction, but also fluid friction, so it is difficult to quantify this torque by theory analysis. Hereby, $T_{m}=5.0e-6N\cdot m$ is regarded as a constant referring from Reference [26].

(12) $$T_{m}=const=5.0e-6N\cdot m$$

The above Equations (6)–(12) constitute the mathematical model of turbine flow sensor.

### 3.2. Verification and Analysis of the Model

**Figure 4:**
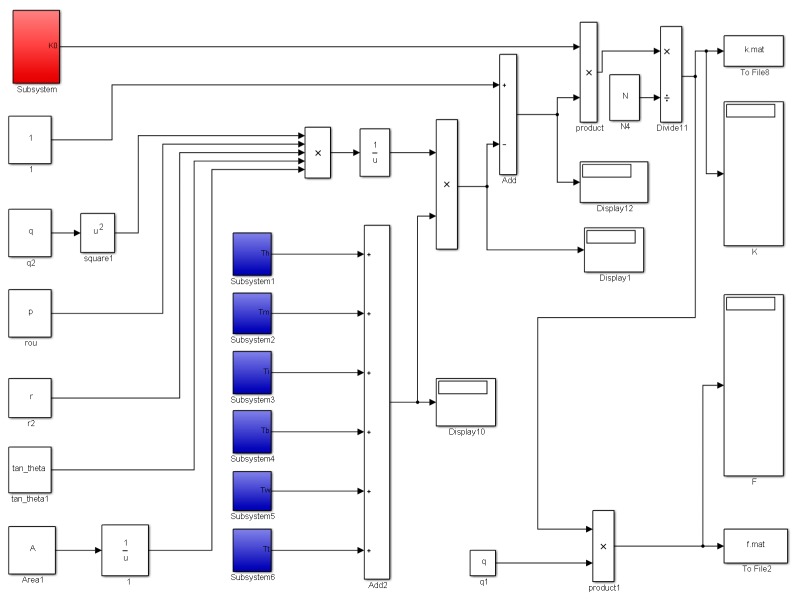
The turbine flow sensor model.

The mathematical model of turbine flow sensor was built in Simulink, which was given in Figure 4. Each resistance torque above was built in the blue modules, which were convenient to check and modify. The variable names were given in the constant modules, and they can be written in a data file(.mat) and loaded into the model. When the turbine structural parameters and the fluid physical parameters are known, the turbine rotational frequency and corresponding meter factor can be calculated using the model. The structural parameters of DN20 turbine flow sensor are shown in Table 2. Water is used as the calculating fluid with a density of 998.2 kg/m^3^, kinematic viscosity of 1.004 × 10^−6^ m^2^/s.

**Figure 5:**
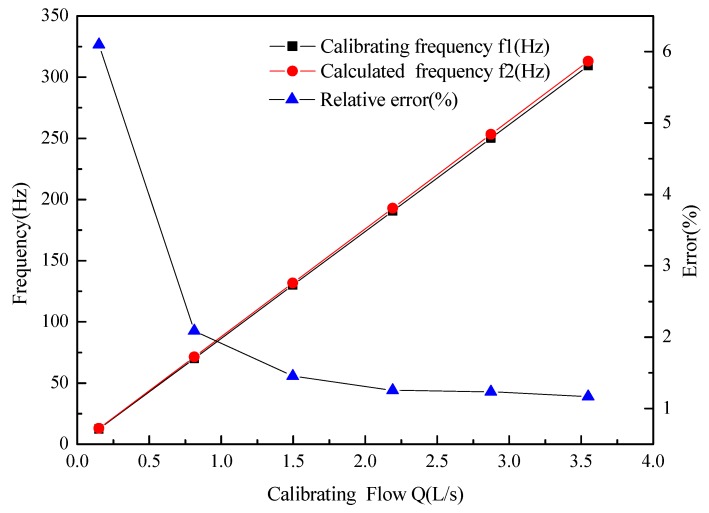
The validation of DN20 Turbine flow sensor model.

The calculated results are shown as Figure 5. Comparing with the calibrating frequency, the maximum relative error of the model is 6.098% in the flow range of 0.15007–3.54868 L/s, which indicates the validity of the model.

### 3.3. Processing in Thelow Frequency Range

Since the calibration frequency range of the electromagnetic turbine flowmeter is 12.30–309.33 Hz, test points within the range satisfy the linearity equation obtained by calibration. However, the frequency points below 12.30 Hz do not meet a linearity relationship. In the mathematical model above, the test points in non-linear range were calculated after removing the electromagnetic resistance torque $T_{m}$, as is shown in Table 3.

According to Table 4, data was segmented into four groups [27]. Each group of data was processed by the least squares method (LSM), correspondingly, four piecewise linear [28,29] flow functions were given in Table 4, in which, the fifth linear equation is given by original experimental calibration. Wide range flow measurement can be realized after programming the equations in software.

## 4. Measurement System and Experiment

### 4.1. Measurement System Realization

**Figure 6:**
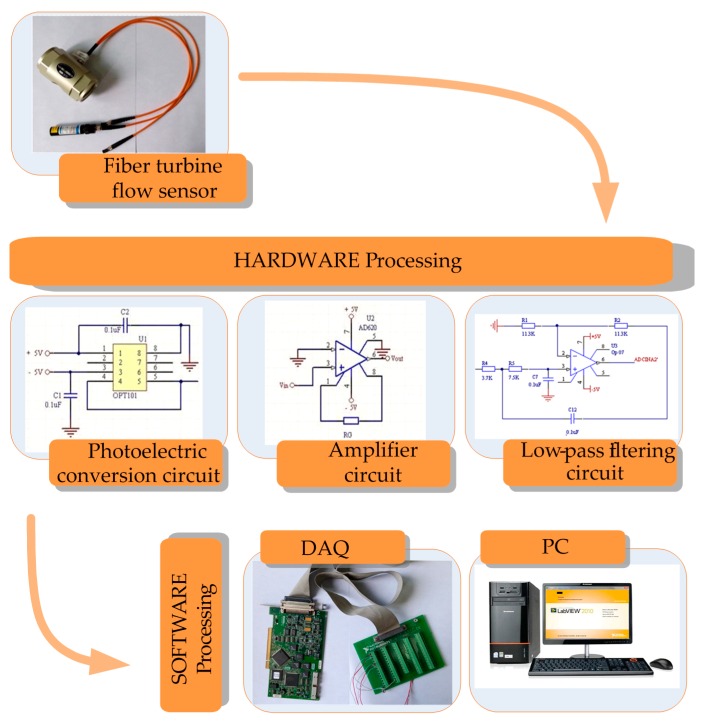
The fiber turbine flow measurement system.

The hardware processing system includes the circuits and the computer measurement system as is shown in Figure 6. The circuits include respectively the photoelectric converter, the amplifier and the filter. The function of the photoelectric converter is to convert the light intensity signals into voltage signals. Due to the small amplifier of the voltage signals and noise signals, the signal filtering should take place after amplifying to save the original signals as much as possible for amplifying without saturation output. If the filtering takes place first, it is possible to introduce some weak interference to make the signals distorted, which is amounts to the noise signals being mixed. The computer measurement system based on the NI DAQCard-6024E card data acquisition and LabVIEW was programmed [30].

Each module of the test system was designed in detail as follows:

- For the photoelectric converter, OPT101 chip produced by Burr-Brown (B-B) was selected. The electric current in the photodiode is proportional to the light intensity. The output voltage of OPT101 increases approximately linearly with the increase of light intensity. Infrared light with a wavelength of 650 nm is used as a light source, when the light with the wavelength of 650 nm is irradiated at the photosensitive area, the output voltage is about the sum of 7.5 mV dark voltage and the voltage responsivity of 0.45 V/μW.
- The operational amplifier is mainly used to improve the signal-to-noise ratio of the input signal and the accuracy of sampling data. Because a small signal is needed to be amplified, the instrument operational amplifier AD620 is used in this system. AD620 is low power consumption and high precision. It has the characteristics of high common mode rejection ratio, wide frequency band, good temperature stability, simple use and low noise. Changing the resistance value of the external resistor, from 1 to 1000 times magnification can be achieved, so it is very suitable for precision amplification of a weak signal. The amplification gain relation of AD620 is shown in the Equation (13), where $R_{G}$ is the Gain resistance.
  (13) $$G=49.4/R_{G}+1$$
- During the optical fiber detection, the noise signals are mainly light source of drift, the environment changes, photodiode noise and the noise of the circuit, etc. The active second-order low-pass filter can filter out high frequency noise signal interferences so that noise signals can obtain rapid and large attenuation. Filtered noise was superimposed on the voltage signal in photoelectric conversion and improved the system SNR to a certain extent. The stable performance general purpose amplifier OP07 was chosen as the op amp of filter, and signals were input from its inverting terminal. The input impedance of the filter is large, the output impedance is very small, its performance is stable and it is easy to adjust.
- NI DAQ-6024E Card is a high-performance E series data acquisition card produced by NI Company, its sampling rate can reach 200 kS/s, and it has 12 bit resolution, 16 single-ended analog input ways. The data acquisition card has a built-in driver, and connects to LabVIEW data acquisition (DAQ) library directly.
- Design of test software was carried out in LabVIEW. Three channel signals are imported into the computer: fiber inner ring voltage, optical fiber outer voltage and electromagnetic flow signal voltage. After dividing the two voltage signals, the output signal was processed by FFT in the module of LabVIEW/Mathscript, then the output frequency was converted into flow rate. All the signals were displayed on the screen. The electromagnetic turbine flow signals were processed in a similar way.

### 4.2. Experimental Results and Discussion

To validate the method of DCC fiber turbine flow measurement, two different tests were carried out with running fluid.

#### 4.2.1. Fiber and Magnetic-Electric Turbine Flow Measurement Contrast Test

**Figure 7:**
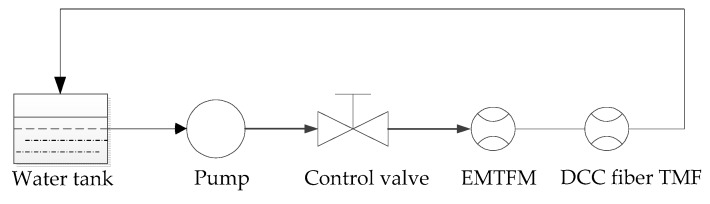
Water flow control loop system schematic diagram.

**Figure 8:**
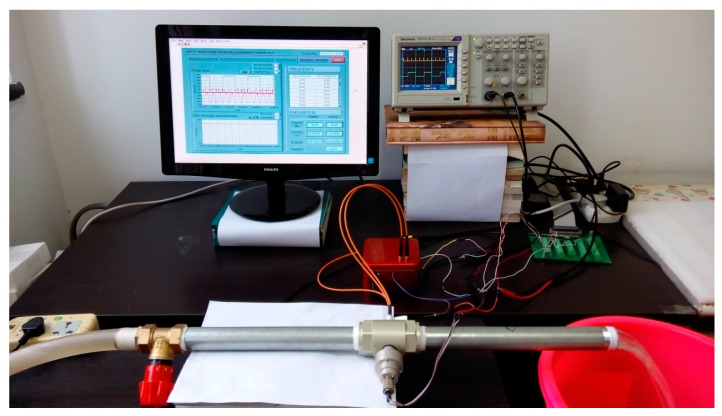
The flow measurement system laboratory test photo.

**Figure 9:**
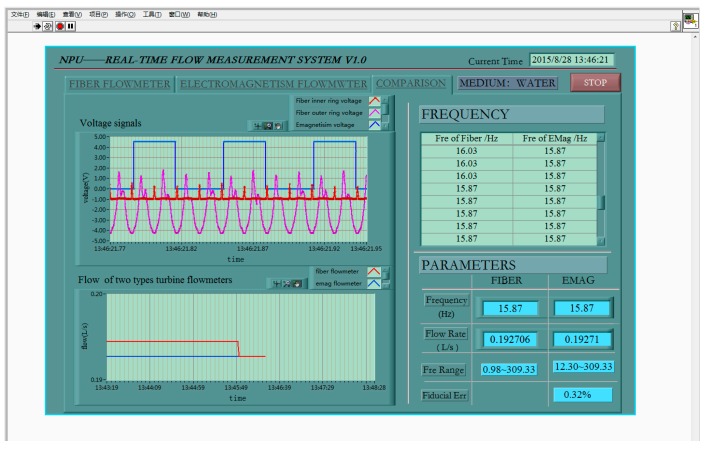
Test display interface of fiber turbine flow sensor contrast with electromagnetic method in the linear range.

In this test, the EMTF was used as a reference flowmeter. Two types of flow sensors measure simultaneously, as is shown in Figure 7, and the laboratory test photo can be seen in Figure 8. The computer test interface is given in Figure 9.

In the linear range of the turbine flow sensor, test data is given in Table 5. It shows that the fiber turbine flow test method is valid and refers accurately to the electromagnetic method. In addition, the resolution of the former is four times bigger than the latter. The reason for this is that the electromagnetic turbine flow sensor output after processing circuit produced by manufacturers is the count of turbine rotations, while the output of fiber sensor is the count of turbine blades. The difference can be seen from the acquisition signals in Figure 9.

#### 4.2.2. Verification Test of Fiber Turbine Flowmeter in Nonlinear Range

**Figure 10:**
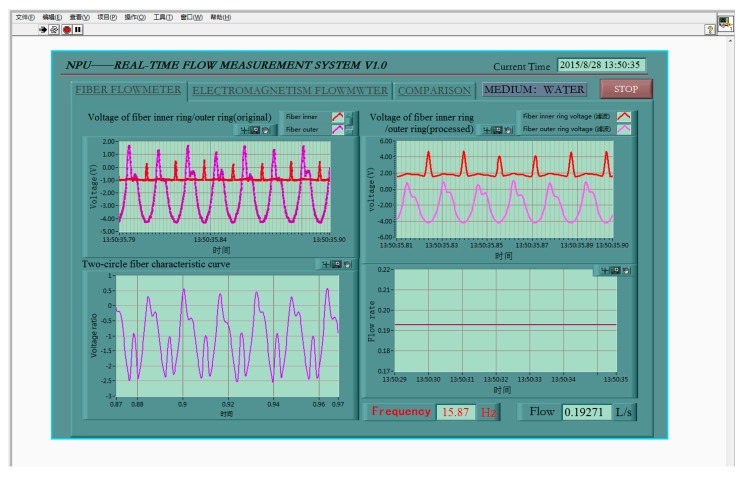
Test display interface of fiber turbine flow sensor in the nonlinear range.

In the nonlinear range of turbine flow sensor, the fiber turbine flow measurement was carried out. The fiber flow test interface is as shown in Figure 10.

Several measuring cups were used to count the cumulative volume flow per minute in the experimental verification. From the data in Table 6, we found that the error increased with the reduction of turbine rotational frequency. When the rotational frequency of the turbine was 4.73 Hz, the error reached 15.42%. Since the experimental calibration method was rough, it had great deviation. In addition, the mathematical model for calculating had also introduced some inaccuracy, but the errors were within an acceptable range, which proved the piecewise linearization was valid. If there had been high-precision calibration devices for calibrating, the measurement accuracy could be improved with the calibrated data reprocessed by piecewise linearity.

The tests above show that the turbine flow measurement range is expanded from 0.15007–3.54868 L/s to 0.0514–3.54868 L/s after using the piecewise linear model, accordingly, the flow range ratio is improved 2.9 times compared with the original range ratio.

## 5. Conclusions and Perspectives

This research mainly contributes a fiber pick-up turbine flow meter for liquid. The DCC fiber probe techniques are well developed in the turbine flowmeter on liquid measurement. To extend the flow range ratio of liquid measurement, the model of turbine flowmeter has been explored. In addition, the flow measurement system has been built and two experiments have been performed in the laboratory. Ultimately, the range ratio of fiber turbine flowmeter increases by nearly three times as much as the original magnetic-electric one. Moreover, the fiber turbine flowmeter can be used in the strong electromagnetic workplace. Additionally, fiber can achieve remote measurement in high temperature situations. Though the results obtained are not so accurate, they are valuable methods for examining the new techniques for liquid measurements under laboratory conditions.

Based on the status quo of applications in turbine flow measurement, the fiber-optical probes techniques also point to future development. This work should be developed further in the following directions:

(1) The optical fiber used for other tests

The quartz optical fiber can resist high temperature and corrosion, and it also has a simple structure, small size, light quality, and easy installation. Based on the principle of light intensity reflecting measurement, it is very suitable for measuring the tip clearance of aircraft engines. Besides, fiber in the turbine flowmeter measures the turbine rotational speed and then is converted into flow rate. Therefore, if the optical fiber probe is mounted to the turbine inner box of aircraft engines, it can be used to measure the rotational speed of aircraft engines.

(2) To measure the mass flow rate

In this paper, the optical fiber turbine flowmeter measured the volume flow rate. If the temperature sensor is integrated, then the density of the fluid can be obtained according to the temperature, and the mass flow rate can be calculated.

(3) Developing multifunctional intelligent instruments of the fiber turbine flowmeter

Using DSP as the core of the digital circuit has powerful functions in terms of storage and operation. The measurement tasks can be programmed and stored, which can realize multi-sensor and multi-parameter measurement. With digital communication interfaces, field instruments based on DSP can realize remote processing in computers via bus interfaces, such as RS232, PIO, CAN, etc.

## Figures and Tables

**Table 1:** The design parameters of the fiber probe.

Fiber Name	Structure Parameter Name	Value
Fiber Axis spacing *d*		300 μm
***Launching fiber***		
Fiber radius $a_{0}$		150 μm
The maximum incidence angle $\theta_{r}$		37°
***Receiving fiber***		
The inner ring	Fiber radius $a_{0}$	150 μm
	The maximum incidence angle $\theta_{c}$	37°
The outer ring A, B	Fiber radius $a_{0}$	150 μm
	The maximum incidence angle $\theta_{c}$	37°

**Table 2:** Structure parameters of DN20 turbine flow sensor.

Parameter Name	Value	Unit	Meaning
r	0.020	m	Inner diameter
N	4	Num.	Leaf blade number
$\bar{r}$	0.0096	m	Leaf blade root mean square radius
θ	50.2827	°	Setting angle of blade
$R_{1}$	0.0003	m	Radius of the shaft
$R_{2}$	0.0004	m	Inner radius of the shaft
$L_{b}$	0.0015	m	Length of friction portion between the shaft and bearing
$L_{h}$	0.0140	m	Axis length of leaf blade
$t_{bt}$	0.0003	m	Thickness of the blade tip
$t_{bh}$	0.0010	m	Blade thickness on the hub
$c_{t}$	0.0220	m	Chord length of the top blade
$R_{t}$	0.0030	m	Radius of the hub
$R_{h}$	0.0095	m	Radius of the turbine
$c_{h}$	0.0130	m	Chord length of the hub
$R_{0}$	0.0100	m	Radius of the pipe

**Table 3:** Frequency ($f_{L}$)-Flow ($q_{L}$) calculating data of fiber turbine flow sensor in the nonlinear range.

$f_{L}$ (Hz)	10.39	9.78	8.55	7.94	6.72	6.11	5.5	4.89	4.28
$q_{L}$ (L/s)	0.1199	0.113	0.0992	0.0923	0.0786	0.0717	0.0649	0.0582	0.0514

**Table 4:** The segment linearization model of fiber turbine flow sensor.

Serial Number	Frequency Range	Flow Rate Equation
1	4.28≤f<5.5	q=0.0111*f+0.0041
2	5.50≤f<6.72	q=0.0112*f+0.0031
3	6.72≤f<8.55	q=0.0113*f+0.0030
4	8.55≤f<10.39	q=0.0112*f+0.0030
5	10.39≤f<12.3	q=0.0158*f−0.0442
6	12.30≤f<309.33	q=0.0114*f+0.0112

**Table 5:** Verification test of the fiber turbine sensor in the linear range.

**State**		1	2	3	4	5	6	7	8
**Electro-Magnetic Test**	**f1(Hz)**	32.36				15.87			
	**q1(L/s)**	0.38119				0.19271			
**Fiber Test**	**f2(Hz)**	32.51	32.36	32.20	32.05	16.03	15.87	15.72	15.57
	**q2(L/s)**	0.38294	0.38119	0.37945	0.37777	0.19445	0.19271	0.19096	0.18922

**Table 6:** Calibration test for fiber flow measurement in the nonlinear range.

State	Verification Test			Fiber Test		Error (%)
	Time (s)	Cumulative Flow (L)	Instantaneous Flow (L/s)	Flow (L/s)	Frequency (Hz)	
1	60	7.76	0.129333	0.11971	10.38	7.44
2	60	6.725	0.112083	0.09909	8.55	11.59
3	60	5.815	0.096917	0.08531	7.33	11.98
4	60	5.415	0.09025	0.07850	6.72	13.02
5	60	4.68	0.07800	0.06657	5.65	14.65
6	60	4.01	0.066667	0.05639	4.73	15.42

## References

[1] Firth J., Ladouceur F., Brodzeli Z., Wyres M., Silvestri L. (2016). A novel optical telemetry system applied to flowmeter networks. Flow Meas. Instrum..

[2] Baker R.C. (1993). Turbine flowmeters: II. Theoretical and experimental published information. Flow Meas. Instrum..

[3] Schena E., Saccomandi P., Silvestri S. (2013). A high sensitivity fiber optic macro-bend based gas flow rate transducer for low flow rates: Theory, working principle, and static calibration. Rev. Sci. Instrum..

[4] Schena E., Massaroni C., Saccomandi P., Cecchini S. (2015). Flow measurement in mechanical ventilation: A review. Med. Eng. Phys..

[5] Takashima S., Asanuma H., Niitsuma H. (2004). A water flowmeter using dual fiber Bragg grating sensors and cross-correlation technique. Sens. Actuators A Phys..

[6] Hu R.P., Huang X.G. (2009). A simple fiber-optic flowmeter based on bending loss. IEEE Sens. J..

[7] Vijayan A., Thakare V., Karekar R.N., Aiyer R.C. (2008). Optical fiber-based macro bend free air flow sensor using a hinge joint: A preliminary report. Microw. Opt. Technol. Lett..

[8] Lim J., Yang Q.P., Jones B.E., Jackson P.R. (2001). DP flow sensor using optical fibre Bragg grating. Sens. Actuators A Phys..

[9] Zhao Y., Chen K., Yang J. (2005). Novel target flowmeter based on differential fiber bragg grating sensor. Measurement.

[10] Lu P., Chen Q. (2008). Fiber Bragg grating sensor for simultaneous measurement of flow rate and direction. Meas. Sci. Technol..

[11] Saccomandi P., Schena E., Silvestri S. (2011). A novel target type low pressure-drop bidirectional optoelectronic air flow sensor for infant artificial ventilation: Measurement principle and static calibration. Rev. Sci. Instrum..

[12] Jiang J.A., Hsu T.Y., Liao W.B., Ouyang C.S. (2009). An Innovative Optical Fiber Flowmeter. J. Chin. Soc. Mech. Eng..

[13] Harun S.W., Yang H.Z., Yasin M., Ahmad H. (2010). Theoretical and experimental study on the fiber optic displacement sensor with two receiving fibers. Microw. Opt. Tech. Lett..

[14] Prelle C., Lamarque F., Revel P. (2006). Reflective optical sensor for long-range and high-resolution displacements. Sens. Actuators A Phys..

[15] Prelle C., Lamarque F., Mazeran P.E. (2002). A new method for high resolution position measurement on long range. J. Eur. Syst. Autom..

[16] Sastikumar D., Gobi G., Renganathan B. (2010). Determination of the thickness of a transparent plate using a reflective fiber optic displacement sensor. Opt. Laser Technol..

[17] Suganuma F., Shimamoto A., Tanaka K. (1999). Development of a differential optical-fiber displacement sensor. Appl. Opt..

[18] Yasin M., Harun S.W., Abdul-Rashid H.A., Kusminarto, Karyono, Ahmad H. (2008). The performance of a fiber optic displacement sensor for different types of probes and targets. Laser Phys. Lett..

[19] Puangmali P., Althoefer K., Seneviratne L.D. (2010). Mathematical Modeling of Intensity-Modulated Bent-Tip Optical Fiber Displacement Sensors. IEEE Tran. Instrum. Meas..

[20] Noshad M., Hedayati H., Rostami A. A proposal for high-precision fiber optic displacement sensor. Proceedings of the Microwave Conference (APMC 2006).

[21] Tang Y., Zhang X., Wang H., Huang Z. (2012). Study on Turbine Flowmeter’s Precision Based on the Variable-Cycle Frequency Measurement. Recent Advances in Computer Science and Information Engineering.

[22] Reddy B.S., Chatterji B.N. (1996). An FFT-based technique for translation, rotation, and scale-invariant image registration. IEEE Trans Image Process..

[23] Thompson R.E., Grey J. (1967). Turbine Flowmeter Performance Model. J. Fluids Eng..

[24] Xu Y. (1992). A model for the prediction of turbine flowmeter performance. Flow Meas. Instrum..

[25] Cheesewright R., Clark C., Cheesewright R., Clark C. (1996). The influence of forces due to electromagnetic pick-ups on the performance of small turbine flowmeters. Proc. Inst. Mech. Eng. Part I J. Syst..

[26] Sun L., Zhou Z., Zhang T. Numerical Simulation of Turbine Flowmeter’s Three-Dimensional Flow Fields. Proceedings of the IEEE the Sixth World Congress on Intelligent Control and Automation (WCICA 2006).

[27] Luxhøj J.T. (1998). An artificial neural network for nonlinear estimation of the turbine flow-meter coefficient. Eng. Appl. Artif. Intel..

[28] Kevenaar T.A.M., Leenaerts D.M.W. (1993). A comparison of piecewise linear model descriptions. IEEE Trans. Circuits Syst. Fund. Theory Appl..

[29] Chen A., Chen C. (2013). Evaluation of Piecewise Polynomial Equations for Two Types of Thermocouples. Sensors.

[30] Cui W., Li B., Chen J., Li X. (2016). A Novel Method of Multi-Information Acquisition for Electromagnetic Flow Meters. Sensors.

